# Trophic amplification of climate warming

**DOI:** 10.1098/rspb.2009.1320

**Published:** 2009-09-09

**Authors:** Richard R. Kirby, Gregory Beaugrand

**Affiliations:** 1 University of Plymouth, School of Marine Science and Engineering, Drake Circus, Plymouth PL4 8AA, UK; 2 The Sir Alister Hardy Foundation for Ocean Science, The Laboratory, Citadel Hill, The Hoe, Plymouth PL1 2PB, UK; 3 Centre National de la Recherche Scientifique, Laboratoire d'Océanologie et de Géosciences' UMR LOG CNRS 8187, Station Marine, Université des Sciences et Technologies de Lille 1 - Lille 1 BP 80, 62930 Wimereux, France

**Keywords:** benthic, cod, plankton, regime shift, temperature, trophic cascade

## Abstract

Ecosystems can alternate suddenly between contrasting persistent states due to internal processes or external drivers. It is important to understand the mechanisms by which these shifts occur, especially in exploited ecosystems. There have been several abrupt marine ecosystem shifts attributed either to fishing, recent climate change or a combination of these two drivers. We show that temperature has been an important driver of the trophodynamics of the North Sea, a heavily fished marine ecosystem, for nearly 50 years and that a recent pronounced change in temperature established a new ecosystem dynamic regime through a series of internal mechanisms. Using an end-to-end ecosystem approach that included primary producers, primary, secondary and tertiary consumers, and detritivores, we found that temperature modified the relationships among species through nonlinearities in the ecosystem involving ecological thresholds and trophic amplifications. Trophic amplification provides an alternative mechanism to positive feedback to drive an ecosystem towards a new dynamic regime, which in this case favours jellyfish in the plankton and decapods and detritivores in the benthos. Although overfishing is often held responsible for marine ecosystem degeneration, temperature can clearly bring about similar effects. Our results are relevant to ecosystem-based fisheries management (EBFM), seen as the way forward to manage exploited marine ecosystems.

## 1. Introduction

Ecosystems exist as dynamic regimes controlled by the interplay among species, the environment and how they interact with external forces such as climate (Scheffer & Carpenter 2003). Many studies have reported the rapid alteration of marine ecosystems throughout the world (Pauly *et al.* 1998; Oguz 2007; Österblom *et al.* 2007). Although human activities and especially fishing are often held responsible for these abrupt ecosystem shifts (Frank *et al.* 2005; Weijerman *et al.* 2005; deYoung *et al.* 2008; Jackson 2008; Casini *et al.* 2009), the oceanic biosphere is now also experiencing rapid global climatic change (Brander 2007). Recently, several studies have described pronounced and sustained responses of marine ecosystems to climate warming (IPCC 2007). Although it has been suggested that marine ecosystems should be resilient to exploitation when managed effectively (Hughes *et al.* 2005) the steady decline of most managed neritic fisheries seems to suggest otherwise (Frank *et al.* 2005). One possible explanation for why ecosystem resource management is problematic could be the difficulty of separating the synergistic effects of fishing from climate (Kirby *et al.* 2009). Indeed, the complexity of living systems has caused some to ask whether ecosystem-based fisheries management (EBFM) in marine ecosystems (Pikitch *et al.* 2004) is even achievable (Longhurst 2006).

The North Sea ecosystem provides 5 per cent of the global fish harvest and has been fished heavily with a particular effect on cod (*Gadus morhua* L.) (Heath 2005). We have previously shown that temperature is more important than wind intensity and direction, salinity, nutrients and oxygen in determining the North Atlantic and North Sea ecosystem dynamic regime (Beaugrand *et al.* 2008). During the 1980s, the North Sea experienced a change in hydro-climatic forcing that caused a rapid, temperature-driven ecosystem shift (Beaugrand & Ibañez 2004). This change in sea surface temperature (SST) altered the plankton and affected the recruitment of cod negatively, at a time when their stocks were also experiencing overfishing (Beaugrand *et al.* 2003; Heath 2005). Changes in the North Sea plankton, following the ecosystem shift, include an increase in microalgae (Kirby *et al.* 2008), a change in the composition and abundance of the holozooplankton (Beaugrand *et al.* 2003), increases in the frequency of jellyfish (Kirby *et al.* 2009) and in the abundance of decapod and echinoderm larvae, and a decrease in bivalve larvae (Kirby *et al.* 2008).

Extensive biological datasets exist for the North Sea that make it uniquely possible to apply an end-to-end ecosystem approach (Travers *et al.* 2007) to investigate the effect of temperature on five trophic levels, primary producers (microalgae), primary, secondary and tertiary consumers (zooplankton, fish and jellyfish), and benthic detritivores (echinoderms and bivalves) (some of these taxa may occupy different trophic levels at different life history stages). These taxa include important commercial species, demersal cod and the benthic flatfish, plaice (*Pleuronectes platessa* L.) and sole (*Solea solea* L.), and incorporate predator–prey interactions that are known to structure pelagic and benthic communities (cod–decapods, Frank *et al.* 2005; decapods–flatfish, van der Veer & Bergman 1987; decapods–bivalves, Barkai & McQuaid 1988). The dataset also contains several taxa whose planktonic larvae are indicators of benthic–pelagic coupling (decapods, echinoderms and bivalves). From this dataset we derived 16 biological descriptors of the North Sea ecosystem that we used to analyse long-term changes for the period 1958 to 2005 by standardized principal component analysis (PCA). To understand the mechanisms underlying the relationship between temperature and ecosystem structure we applied causal modelling (Legendre & Legendre 1998); this is a statistical method that can be used to determine the type of control in an ecosystem (bottom-up or top-down; Cury *et al.* 2003). In this way, we probed much deeper than current proposed approaches (Cury *et al.* 2003). Our results show that temperature has been an important driver of North Sea trophodynamics for nearly 50 years and that a recent change in temperature has established a new ecosystem dynamic regime by modifying the strength and direction of some trophic interactions.

## 2. Material and methods

### (a) Biological data

**Figure 1.**
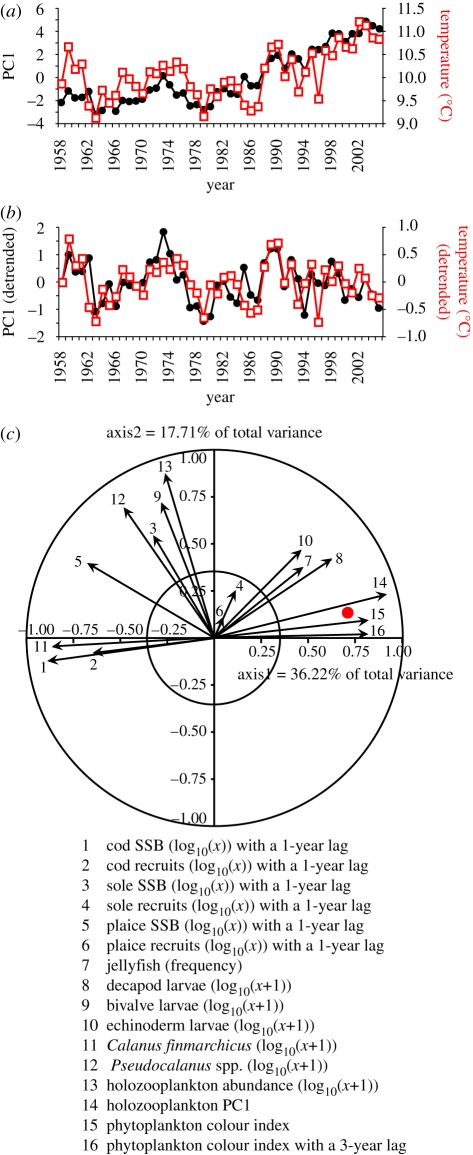
Principal component analysis on long-term changes in the North Sea ecosystem. (*a*) Relationship between the first principal component (36.22% of the total variance) in black dots and annual sea surface temperature (SST) in red squares. (*b*) Relationship between the detrended first principal component and detrended annual SST. (*c*) Normalized eigenvectors 1 and 2 (53.93% of the total variance); both circle of correlation (outer circle) and circle of equilibrium (inner circle) descriptor contribution (*c* = 0.353) are displayed. Variables inside the latter circle have a non-significant contribution. Annual SST (red dot) is a supplementary variable and does not contribute to the principal components. A total of 16 biological variables were included in the analyses. A three-year lag was introduced into the phytoplankton colour index due to the positive correlation that exists between this variable and decapod larval abundance (Kirby *et al.* 2008).

Plankton data were collected by the Continuous Plankton Recorder (CPR) survey. The CPR survey has operated in the North Sea on a routine monthly basis since 1946. Seawater enters the CPR through a front aperture and the plankton is retained on a moving band of silk gauze of mesh size 270 µm that is slowly wound into a tank of formalin. In the laboratory the silk gauze is cut into sections (a CPR sample), each representing the plankton from 3 m^3^ of water taken during 10 nautical miles (18 km) of tow at an average depth of 7 m. Up to 450 taxa are identified and enumerated. The methods of CPR sampling and analysis have remained consistent throughout the time series (Batten *et al.* 2003). The plankton data we used comprise the abundance of the holozooplanktonic copepods *Calanus finmarchicus* and *Pseudocalanus* spp., a measure of the total holozooplankton, and the abundance of the merozooplanktonic larvae of decapods, echinoderms and bivalves. The dataset also includes the frequency of jellyfish material estimated by nematocyst frequency. An index of holozooplankton composition was also created using a standardized PCA. An annual mean was first calculated for all holozooplanktonic species or taxonomic groups. Then, species or taxonomic groups with an annual relative abundance >0.001 and a presence >30 per cent for all years in the period 1958 to 2005 were selected. This procedure allowed the selection of 35 holozooplankton species or taxonomic groups. Abundance data in the matrix (48 years × 35 species or taxonomic group) were transformed using the function log_10_(*x* + 1). A PCA was then performed on the correlation matrix (35 × 35 species) to identify the main pattern of long-term changes in holozooplankton community structure (examination of principal components). An estimate of the amount of chlorophyll in each CPR sample was derived from the phytoplankton colour index that measures the greenness of the silk due to both trapped cells and fragile phytoplankton taxa that burst upon impact. The phytoplankton colour index correlates well with both fluorometer and satellite measures of chlorophyll (Raitsos *et al.* 2005). Data on North Sea demersal fish (cod, plaice and sole spawning stock biomass (SSB) and recruits) were obtained from http://www.ices.dk and are derived from a virtual population analysis based on fisheries data. We restricted our analysis to commercially fished demersial species, because, with the exception of the herring (*Clupea harengus*), similar long-term datasets for small pelagic species such as sandeel (*Ammodytes marinus*) and sprat (*Sprattus sprattus*) do not exist and to shorten our time series to accommodate these species would have limited the power of causal modelling. From our plankton and fish dataset we derived 16 biological descriptors of the North Sea ecosystem (figure 1).

### (b) Physical data

Annually averaged sea surface temperature data for the North Sea were calculated from the COADS 1-degree enhanced dataset provided by the comprehensive NOAA-CIRES Climate Diagnostics Center Database (Boulder, Colorado, USA) (Woodruff *et al.* 1988).

### (c) Statistical analysis

All plankton abundance data were first transformed using the function, log_10_(*x* + 1). Fish data were transformed using the function, log_10_(*x*). Long-term changes in the ecosystem state were first studied by means of principal components. Relationships between indices of ecosystem change (e.g. principal components) and SST were then investigated by correlation analysis. Correlation analyses were performed on both original and detrended data to examine the relationships between temperature and ecosystem change more closely (figure 1*a*,*b*). A one-year lag was introduced when the correlations were calculated between fish data (SSB and recruits) and plankton or SST. A three-year lag was introduced between decapod larvae and the phytoplankton colour index (Kirby *et al.* 2008). The time series were detrended by means of singular spectrum analysis (SSA) (Vautard & Ghil 2002). This method uses a principal component analysis performed on an autocovariance matrix (also called a Toeplitz matrix) to divide a time series into a succession of signals of decreasing variance (Beaugrand & Reid 2003). The method of SSA is also known both as eigenvector filtering (EVF) and PCA of processes. SSA decomposes a signal into different components (long-term trends, cycles or pseudocycles and year-to-year variability) and it has been applied usefully in marine ecology before (Ibanez & Etienne 1992; Molinero *et al.* 2005). Detrended time series were calculated by subtracting from each original time series its respective long-term trend identified by the first principal component of the SSA. Probabilities were calculated with consideration to a modified temporal Box–Jenkins autocorrelation function (Chatfield 1996) with adjusted degrees of freedom (Chelton 1984). Relationships between each biological descriptor and PC1 were assessed by examining the eigenvectors (figure 1*c*).

**Figure 2.**
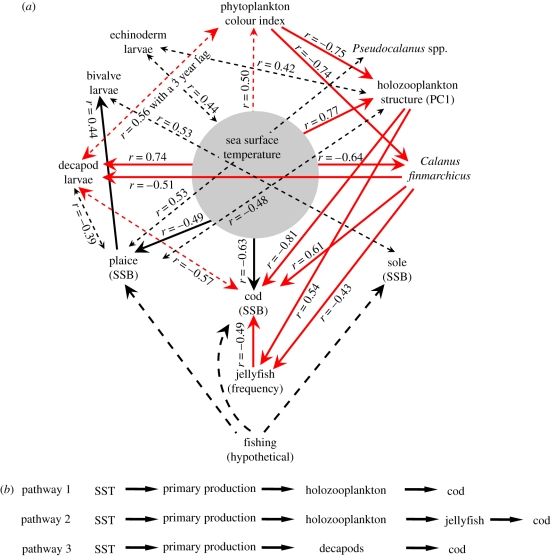
Statistical causal modelling to determine the pathways by which temperature propagates through the North Sea food web. (*a*) Causal model based on the examination of both ordinary and first-order partial coefficients of correlation between 12 variables. Only significant ordinary coefficients of correlation (*p* < 0.05) are indicated on the diagram. Unidirectional solid arrows indicate a statistical causal link between variables with an indication of direction. Bidirectional dashed arrows indicate a correlation between variables without direction. Red arrows indicate pathways contributing to the trophic amplification of temperature effects with respect to cod. Note that the red arrow between sea surface temperature (SST) and phytoplankton is shown dashed as the analysis did not indicate direction in this case. However, we consider it unlikely that phytoplankton influences SST over the timescale of our study. Black, unidirectional, dashed arrows suggest the top-down effects of fishing. (*b*) Three indirect pathways leading to the trophic amplification of temperature on cod.

The linear coefficients of first-order partial correlation were then calculated to determine how a climatic signal propagates into the food web, and the results presented synthetically on a global diagram (figure 2*a*). The partial correlation coefficients allow the relationship between two variables to be measured after removing the effect of a third variable while keeping its mean constant. The partial correlation coefficient between variables 1 and 2, removing the linear effect of variable 3 (noted *r*_12.3_), was calculated using the equation provided by Legendre and Legendre (1998).
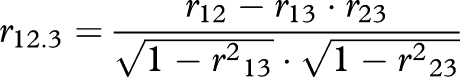
where *r*_12_, *r*_13_ and *r*_23_ are simple linear coefficients of correlation between 1 and 2, 1 and 3, and 2 and 3, respectively. A simple way of studying statistical causal relationships may be carried out on three variables by looking at both ordinary and partial correlation coefficients (Legendre & Legendre 1998). Using four reference models (see figure S1 in the electronic supplementary material), we determined causal relationships between variables by applying the technique to each possible triplet of variables. By calculating both the ordinary correlation and the first-order partial correlation coefficients, causal modelling allows for the detection of spurious correlations, which is an important feature of the method. For example, in electronic supplementary material figure S1, model 2, the ordinary correlation between variables 2 and 3 can be found to be significant, because variable 1 influences both variable 2 and variable 3. By calculating the first-order partial coefficient of correlation (*r*_23.1_) this effect can be detected. Because causal modelling adds other criteria to validate the model, such as the interaction factor *r*_12_ × *r*_13_ = *r*_23_, spurious correlations are minimized.

**Figure 3.**
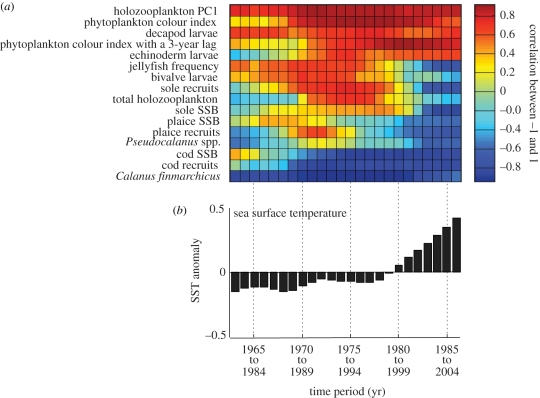
Examination of the effect of temperature on the relationships among species and trophic levels for the period 1958 to 2005. (*a*) Sliding correlation analysis between the first principal component and the 16 biological descriptors of the North Sea ecosystem using a time window of 20 years. (*b*) Sliding average of annual sea surface temperature (SST) using a time window of 20 years. Each vertical bar represents a 20 year period; for example, the first three vertical bars represent the overlapping periods 1963 to 1982, 1964 to 1983, and 1965 to 1984, respectively. Consequently, in the axis labels the upper and lower years denote the beginning and the end of the time period, respectively.

**Figure 4.**
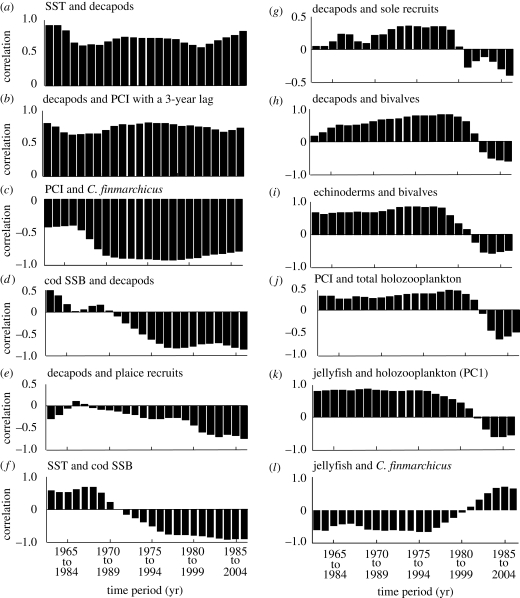
Sliding correlation analysis between pairs of variables for the period 1958 to 2005. (*a*) sea surface temperature (SST) and decapods. (*b*) Decapods and phytoplankton colour index (PCI) with a three-year lag. (*c*) PCI and *C. finmarchicus*. (*d*) Cod SSB and decapods. (*e*) Decapods and plaice recruits. (*f*) SST and cod SSB. (*g*) Decapods and sole recruits. (*h*) Decapods and bivalves. (*i*) Echinoderms and bivalves. (*j*) PCI and total holozooplankton. (*k*) Jellyfish and holozooplankton (PC1). (*l*) Jellyfish and *C. finmarchicus*. Each vertical bar represents a 20 year period, for example, the first three vertical bars represent the overlapping periods 1963 to 1982, 1964 to 1983, and 1965 to 1984, respectively. Consequently, in the axis labels, the upper and lower years denote the beginning and the end of the time period, respectively.

Finally, sliding correlation analysis was applied to assess the temporal stability of the relationships among variables for a time window of 20 years. This approach calculates correlations for moving time periods with an increment of one year. As the technique is sensitive to the chosen time period, windows were examined ranging between 10 and 25 years; these all gave similar conclusions. First, the technique was applied between the first principal component and the 16 biological variables (figure 3). Second, the technique was used on selected pairs of variables (figure 4).

## 3. Results

The PCA performed on the table of 16 biological indicators of the North Sea ecosystem suggested a strong influence of temperature on the ecosystem dynamic regime (figure 1*a*) (*r* = 0.74, *p* = 0.002, probability corrected for temporal autocorrelation) as indicated by the first principal component (36.22% of the total variance). This positive link is still visible when both time series are detrended (*r* = 0.59, *p* = 0.0001, probability corrected for autocorrelation; figure 1*b*). Examination of the eigenvectors shows that the influence of temperature pervades the whole ecosystem (figure 1*c*).

Causal modelling revealed that temperature acts through several direct and indirect pathways (figure 2*a*) to influence the trophodynamics of the North Sea ecosystem. A total of five direct (statistical) causal links between SST and biological parameters were identified (holozooplankton structure, abundance of *Calanus finmarchicus*, cod SSB, plaice SSB and decapod larvae) and two correlations between SST and the phytoplankton colour index and echinoderm larvae. Taking cod as an example of the complexity of the system, we see that temperature acts both directly (negative effect) and indirectly via the food web (negative effects). This can be summarized in the following way:
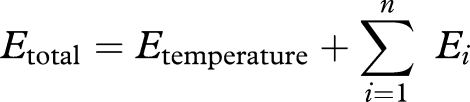
where *n* is the number of considered species, *E*_total_ the total influence of temperature on the target species (e.g. cod), *E*_temperature_ the direct influence of temperature on the target species and *E_i_* the influence of temperature through the food web.

Indirect pathways include (i) the effects of temperature on the zooplankton prey of larval cod (figure 2*b*, pathway 1), (ii) through decapods (figure 2*b*, pathway 3), which may operate through decapod predation on holozooplankton (Frank *et al.* 2005), and (iii) through predation by jellyfish (figure 2*b*, pathway 2). For example (see figure S1, model 4 in the electronic supplementary material), in the case of SST (variable 1), the preferred prey of cod, *C. finmarchicus* (variable 2) and cod (variable 3), both the ordinary coefficients of correlation are significant (*r*_12_ = −0.64 (*p*_12_ < 0.0001); *r*_13_ = −0.63 (*p*_13_ < 0.0001); *r*_23_ = 0.61 (*p*_23_ < 0.0001)) and the first-order partial coefficients of correlation are significant (*r*_12.3_ = −0.50 (*p*_12.3_ = 0.0007); *r*_13.2_ = −0.36 (*p*_13.2_ = 0.02); *r*_23.1_ = −0.31 (*p*_23.1_ = 0.04)). Therefore, the negative influence of temperature on cod is direct and also indirect through its negative influence on *C. finmarchicus*, which is positively related to cod.

When sliding correlation analysis was applied between the first principal component (figure 1*a*) and each of the 16 biological variables with a moving window of 20 years, the relationship between the first principal component and some variables was found to be constant (figure 3*a*), for example, *C. finmarchicus* and cod recruits (both negatively correlated with the first principal component). Other biological variables in the North Sea ecosystem appear to either develop through time (e.g. echinoderm larvae), or show a change in the sign of their relationship after the beginning of the 1980s (e.g. jellyfish frequency) (figure 3*a*). The changes in these variables coincide with a rapid and sustained increase in temperature (figure 3*b*).

A pairwise analysis of the relationships among biological variables reveals three types of interaction (figure 4). The first is a constant interaction over time (i.e. at least the time period considered); this is shown by the relationships between SST and decapod larvae (figure 4*a*), decapod larvae and the phytoplankton colour index with a three-year-lag (figure 4*b*), and *C. finmarchicus* and phytoplankton colour (figure 4*c*), for example. The second type of interaction is where a significant relationship develops such as the negative relationship between cod SSB and decapod larvae (figure 4*d*). A strengthening negative relationship also develops between plaice and their decapod predators (figure 4*e*). The third type of interaction is where the sign of the relationship reverses (figure 4*f–l*).

## 4. Discussion

Temperature, through its effect on physiology, can modulate species distributions, interactions and trophodynamics (Cury *et al.* 2008). Unfortunately, there are few ecosystems where sufficient data exist to examine the effect of temperature across several trophic levels and long timescales. The North Sea dataset used in this study is unique in this respect. Our results show that temperature has been an important mechanism (figure 1) driving the trophodynamics (figure 2) of this heavily fished marine ecosystem for nearly 50 years. A recent pronounced change in temperature (figure 3) appears to have established a new ecosystem dynamic regime through a series of internal mechanisms (figure 4). Our analysis of the North Sea ecosystem indicates that the influence of temperature translates through the food web to the ecosystem level. It is, therefore, unsurprising that climate change is held responsible for several recent abrupt ecosystem shifts, especially in the marine environment where the plankton food web is especially sensitive to hydroclimatic change (Beaugrand *et al.* 2003).

Positive correlations between ecosystem dynamic regimes and the hydroclimatic environment have been reported before (Weijerman *et al.* 2005), but until now it had remained unclear how temperature might propagate through the ecosystem to affect energy allocation, predator–prey interactions and benthic–pelagic coupling (Cury *et al.* 2008). By using causal modelling we have revealed how the food web can propagate the effect of temperature through a number of indirect pathways. We call this intensification of the effect of temperature through indirect pathways a trophic amplification. Trophic amplification represents an alternative to positive feedback as a mechanism by which abrupt ecosystem shifts occur (Scheffer & Carpenter 2003). It is also distinct to true positive feedback, because, in our case, we have not considered any possible influence of plankton on climate (Lovelock 1979; Charlson *et al.* 1987). At the level of the ecosystem, trophic amplification intensifies the effect of climate warming, potentially leading to a new attractor (ecosystem dynamic regime) (Scheffer & Carpenter 2003). This could explain the often nonlinear and sometimes unpredictable response of ecosystems to climate variability (Muradian 2001; Taylor *et al.* 2002; Hsieh *et al.* 2005). As a top predator, cod seem particularly vulnerable to trophic amplification of the effects of temperature (figure 2*b*). Even though certain conditions might seem to favour cod, such as the increase in decapod prey in the benthos (Kirby *et al.* 2008, 2009), the amplified effect of temperature, especially on the larval stage (a critical phase in the life cycle of fish; Cushing 1990), is overwhelming.

Rapid shifts between alternative attractors occur in lakes when temperature affects the development of the clear-water phase to modify the plankton food web (Scheffer & Carpenter 2003). A sliding correlation analysis revealed that there was a differential response among biological variables to a pronounced change in temperature; some pairs of variables were found to be constant, whereas others developed or showed a change in the sign of their relationship (figure 3). This differential response is a clear manifestation of the effects of the North Sea abrupt ecosystem shift (Weijerman *et al.* 2005) on the trophodynamics of the system. This analysis also revealed that the main component of the food web, with respect to North Sea cod (the copepod, *C. finmarchicus*; Beaugrand *et al.* 2003), is stable through time. Consequently, a single causal modelling (figure 2*a*) for the whole time period was sufficient to understand the effects of the trophic amplification of temperature in the food chains involving cod.

Although we did not include the effect of fishing in our analyses (we found that the effect of cod fishing mortality on the first principal component was not significant, *r* = 0.55, *p* = 0.197), the removal of top predators can also alter ecosystems by triggering trophic cascades (Pace *et al.* 1999), and in pelagic marine ecosystems, overfishing is considered a likely cause (Daan *et al.* 2005; Frank *et al.* 2005; Scheffer *et al.* 2005; Casini *et al.* 2008). Our time series begins around the time of the disappearance of the high trophic level Atlantic bluefin tuna (*Thunnus thynnus*) from the North Sea in the early 1960s; prior to this, from 1900 to 1950, there was a commercial tuna fishery in the region (MacKenzie & Myers 2007). Consequently, the trophic hierarchy of the North Sea ecosystem was altered near the start of our time series. Cod have also been fished heavily in the North Sea (Heath 2005), and where this has occurred elsewhere, it has been held responsible for alterations in other trophic components of the ecosystem (Frank *et al.* 2005), especially when coupled with unfavourable recruitment conditions for cod (Casini *et al.* 2009). Although temperature appears an important driver of both plankton and cod SSB in the North Sea, overfishing may nevertheless have modulated the ecosystem dynamic regime through the interaction between bottom-up and top-down effects in the plankton (Stige *et al.* 2009). In particular, fishing may have enabled an increase in gelatinous plankton in the North Sea, as occurred in the Black Sea (Daskalov *et al.* 2007), with consequent effects on both plankton and fish recruitment (figure 2*a*; Kirby *et al.* 2009).

The pairwise analysis of the relationships among biological variables showed three types of interaction in the ecosystem (figure 4). The first, such as the relationship between decapod larvae and SST (figure 4*a*), and the relationship between decapod larvae and phytoplankton colour index with a three-year lag (figure 4*b*) was constant through time and has been seen before (Kirby *et al.* 2008). These relationships are likely to reflect the positive influence of temperature and food on decapod reproductive output and larval survival (Kirby *et al.* 2008). Although the relationship between larval abundance and the size of benthic populations has not been generally established for invertebrate macrofauna, larval surveys are a long established means of estimating the spawning stocks of decapods (Briggs *et al.* 2002). The increase in decapod larval abundance in CPR samples reflects changes in larval numbers of Polybiinae (swimming crab larvae), *Upogebia deltaura*, *Callianassa subterranea* and *Cancer pagurus* (Rees 1955; Lindley *et al.* 1993), predominantly. Fisheries data show that landings of the predatory decapods *Pandalus* and *Nephrops*, (ICES 2006) and *Cancer* (Heath 2005) have increased markedly in the North Sea, and Rees *et al.* (2007) also noted increases in *U. deltaura* and *C. subterranea* in certain regions.

The second interaction we observed is where a significant relationship develops through time, such as the negative relationship between cod SSB and decapod larvae (figure 4*d*), which may indicate the opposite influences of temperature on both cod recruitment (Beaugrand *et al.* 2003) and decapod larval abundance (Kirby *et al.* 2008), and reduced predation by adult cod on decapods (Frank *et al.* 2005). A strengthening negative relationship also develops between both plaice and their decapod predators (figure 4*e*), which may reflect increased decapod numbers and predation on plaice recruits (van der Veer & Bergman 1987); this may also explain the change in the relationship between decapods and sole recruits (figure 4*g*, see below). Interestingly, this suggests that different fisheries may be linked through changes in the food web. However, it is the third type of interaction, where the sign of the relationship reverses around an ecological threshold (figure 4*f–l*), that is perhaps the most interesting, as it reveals nonlinearity in the system. For example, the relationship between cod SSB and temperature is positive during the cold-water phase (1962–1982) of the North Sea and becomes negative during the warm-water phase (1989 onwards; Beaugrand 2004; figure 4*f*). The effect of temperature on cod may reflect the thermal tolerance of the preferred prey of larval cod, for example, *C. finmarchicus* (figure 2; Beaugrand *et al.* 2003). These results re-emphasize how internal processes may amplify a small change in an external driver, such as a 1°C temperature change (Beaugrand 2004), to overcome an ecological threshold and lead to a new attractor.

The effect of temperature may have also influenced a number of internal processes such as predator–prey interactions. Examples of changes in interspecific relationships include the change in the sign of the relationships between decapods and sole (figure 4*g*) and between decapods and bivalves (figure 4*h*), which may reflect increased predation by decapods in the benthos (van der Veer & Bergman 1987; Barkai & McQuaid 1988; Kirby *et al.* 2008). Like decapods, the larval production of benthic echinoderms is also affected positively by North Sea temperature (Kirby *et al.* 2008) and so it is unsurprising that the trend between echinoderms and bivalves (figure 4*i*) is similar to that between decapods and bivalves (figure 4*h*). Numbers of the dominant echinoderm in CPR samples, the psammivorous detritivore, *Echinocardium cordatum* (Kirby *et al.* 2007), have also increased in the benthos (Rees *et al.* 2007). Changes in the relationships between microalgae and holozooplankton (figure 4*j*), and between jellyfish and holozooplankton PC1 (figure 4*k*) could reflect trophic interactions similar to those suggested to have occurred in other ecosystems (Frank *et al.* 2005; Daskalov *et al.* 2007). The opposite relationships between jellyfish and holozooplankton PC1, and jellyfish and *C. finmarchicus* (figures 2*a* and 4*k*,*l*) can be explained by the strong negative relationship that exists between these two zooplankton components. Holozooplankton PC1 represents smaller zooplankton than *C. finmarchicus*, which show opposite patterns of long-term change due to the influence of temperature (figure 1*c*). Taken together, these alterations in the strength and sign of the relationships among taxa are a clear manifestation of nonlinear dynamics in an ecosystem (Muradian 2001; Scheffer & Carpenter 2003).

In the North Sea the new dynamic regime favours jellyfish in the plankton and decapods and detritivores (echinoderms) in the benthos (Kirby *et al.* 2008, 2009). Although fishing down marine food webs has previously been held responsible for their degeneration (Pauly *et al.* 1998), temperature can clearly bring about the same effect. So far, the North Sea has only experienced a 1°C change in SST and this is expected to double by the end of the century (Scenario A2, IPCC 2007). The societal demand for managing marine resources compels us to increase our efforts to understand the complexities of the ecosystem. Our results show that bottom-up control is an important influence on a marine ecosystem and, in the case of the North Sea, on the important commercial species, cod (direct and indirect effects), plaice and sole (indirect effects), and so climate change should be considered along with the effect of fishing in EBFM. The nonlinear dynamics and the complexity of possible trajectories among components of the ecosystem simply make the application of EBFM more challenging (Longhurst 2006; deYoung *et al.* 2008).
